# Comparing counselling alone versus counselling supplemented with guided use of a well-being app for university students experiencing anxiety or depression (CASELOAD): protocol for a feasibility trial

**DOI:** 10.1186/s40814-016-0119-2

**Published:** 2017-01-23

**Authors:** Emma Broglia, Abigail Millings, Michael Barkham

**Affiliations:** 1 Department of Psychology, University of Sheffield, Sheffield, S10 2TN UK; 2 Centre for Psychological Services Research, Department of Psychology, University of Sheffield, Sheffield, S10 2TN UK

**Keywords:** Student mental health, Anxiety, Depression, Counselling, Well-being app, Behaviour monitoring, Augmented therapy, Feasibility, Acceptability

## Abstract

**Background:**

University counselling services face a unique challenge to offer short-term therapeutic support to students presenting with complex mental health needs and in a setting which suits the academic timetable. The recent availability of mobile phone applications (apps) offers an opportunity to supplement face-to-face therapy and has the potential to reach a wider audience, maintain engagement between therapy sessions, and enhance therapeutic outcomes. The present study, entitled Counselling plus Apps for Students Experiencing Levels of Anxiety or Depression (CASELOAD), aims to explore the feasibility of supplementing counselling with guided use of a well-being app.

**Methods/Design:**

Forty help-seeking university students (aged 18 years and over) with symptoms of moderate anxiety or depression will be recruited from a University Counselling Service (UCS) in the United Kingdom (UK). Participants will be recruited via counsellors who provide the initial clinical assessment and who determine treatment allocation to one of two treatments on the basis of client-treatment fit. The two conditions comprise (1) counselling alone (treatment as usual/TAU) or (2) counselling supplemented with guided use of a well-being app (enhanced intervention). Trained counsellors will deliver up to six counselling sessions in each treatment arm across a 6-month period, and the session frequency will be decided by client-counsellor discussion. Assessments will occur at baseline, every counselling session, post-intervention (3 months after consent) and follow-up (6 months after consent). Assessments will include clinical measures of anxiety, depression, psychological functioning, specific mental health concerns (e.g. academic distress and substance misuse), resilience and therapeutic alliance. The usage, acceptability, feasibility and potential implications of combining counselling with guided use of the well-being app will be assessed through audio recordings of counselling sessions, telephone interviews with participants, focus groups with counsellors and counsellor notes.

**Discussion:**

This study will inform the design of a randomised pilot trial a﻿nd﻿ a definitive trial which aim to improve therapy engagement, reduce dropout and enhance clinical outcomes of student counselling.

**Trial registration:**

ISRCTN55102899

**Electronic supplementary material:**

The online version of this article (doi:10.1186/s40814-016-0119-2) contains supplementary material, which is available to authorized users.

## Background

There is limited evidence demonstrating the effectiveness of counselling services in higher education (HE), and recent government initiatives have negatively impacted on student services. These changes have been particularly noticeable in the United Kingdom (UK), since new policies have raised tuition fees and widened access to university without financially supporting service growth [1]. As a result, there is more pressure on university counselling services (UCSs) to demonstrate effectiveness and explore innovative solutions to continue to offer high-quality support with less resource in a sustainable way [2]. This is particularly challenging for student counselling services because they support a unique population with mental health needs that require counsellors/therapists who are trained and experienced in the academic context. For example, students require support that fits within the academic calendar and around periods of time when students are away from campus. Technology-assisted therapy provides a promising solution to support student counselling, but the feasibility and effectiveness of doing so are unknown [3, 4]. The current study aims to address these challenges by exploring the feasibility of supplementing face-to-face counselling with guided use of a well-being app for university students experiencing anxiety or depression.

### University counselling services (UCS) in the UK

UCSs are frequently evolving to address student demands and this has been widely accepted as a necessity [5, 6]. For example, a recent qualitative study summarised the changes experienced in a UK UCS [7]. These included the following prominent themes: (1) counsellors are being encouraged to work more flexibly by catering the number and frequency of therapy sessions to best suit their clients’ needs; (2) UCSs are offering more online support (e.g. online self-help) to manage growing demands with limited financial resources; (3) counsellors’ workloads are increasing to maintain high standards and meet growing demands in the absence of service expansion; (4) UCSs continue to be pressured to demonstrate effectiveness; and (5) there are concerns that UCSs may not be collecting the right type of data, not using the available data, or missing data after counselling. Furthermore, a recent investigation of the usage and acceptability of therapeutic technology in student counselling revealed that many services are interested in knowing how contemporary therapeutic technology, such as mobile apps, can be used in student services and the potential implications of doing so (Broglia, Millings, & Barkham: The burden of student mental health on embedded counselling services in UK Higher and Further Education institutions, submitted). Taken together, these findings demonstrate how UK UCSs are embracing change and exploring innovative solutions to address recent trends in student mental health.

### Feedback in therapy

In conjunction with finding new innovative solutions to address changes in UCSs, it is also important to explore whether existing methods can be enhanced to improve outcomes. One such method involves therapists providing feedback to clients about their responses on a clinical outcome measure in order to help clients acknowledge their progress or to raise discussion about adapting treatment. This method of integrating feedback into therapy has been widely explored and its impact on clinical outcomes have been summarised in a recent scoping review [8]. For example, compared to clients who received no feedback, clients who had feedback from clinical measures discussed in therapy (i) improved to a greater degree, (ii) demonstrated improvements sooner, (iii) required fewer therapy sessions, (iv) were less likely to drop-out of therapy and (v) maintained improvements at 6- and 12-month follow-ups. However, by contrast, a recent meta-analysis of 17 clinical trials found no significant differences between feedback and no-feedback groups on symptom outcomes [9]. Whilst there are mixed findings on the potential benefits of using feedback in therapy, the meta-analysis also concluded that the clinical trials exploring feedback in therapy exhibit strong bias and weak methodology.

Taken together, these mixed findings highlight the need for more rigorously designed clinical trials to explore the potential benefits of discussing feedback in therapy. Additionally, there is a need to understand the potential of augmenting therapy using technology, and monitoring feedback is a logical area in which to do this [10], given the ease with which mobile devices enable individuals to track various aspects of self-related data. Aside from the use of mobile technology to increase access to and uptake of psychological support, research demonstrates that therapeutic technologies can be cost-effective, acceptable, and show the potential to enhance the therapeutic process [11–13]. Therefore, whilst the positive findings regarding feedback relate to clinical outcome, the current study aims to apply the feedback model to discussing client thoughts, behaviours, emotions and activities monitored daily on a well-being app.

### The current study

The primary aim of the current study, offering face-to-face counselling, is to demonstrate whether discussion and guided use of a well-being app can be integrated into counselling sessions with university students experiencing anxiety or depression. This feasibility metric will be assessed through evaluation of therapeutic discussion from counselling audio recordings, telephone interviews with participants and a focus group with counsellors. Through this primary aim, the current study will also evaluate feasibility factors related to recruitment, acceptability, intervention delivery and clinical outcome monitoring. Therefore, the primary feasibility outcomes will comprise (i) recruitment duration to reach target sample size, (ii) client treatment preferences, (iii) acceptability of randomisation, (iv) intervention fidelity, (v) client/counsellor satisfaction and (vi) completion rate of follow-up measures. Secondary aims will explore the preliminary impact (in terms of effectiveness) and potential moderators for a fully powered definitive RCT. Preliminary impact will be assessed by comparing differences in therapeutic alliance between TAU and the enhanced intervention condition, as well as capturing participants’ views on the impact counselling has had on their well-being and ability to cope at university. Potential intervention moderators will include app usage during and between counselling sessions, as well as client characteristics.

## Methods/Design

### Study design

**Fig. 1:**
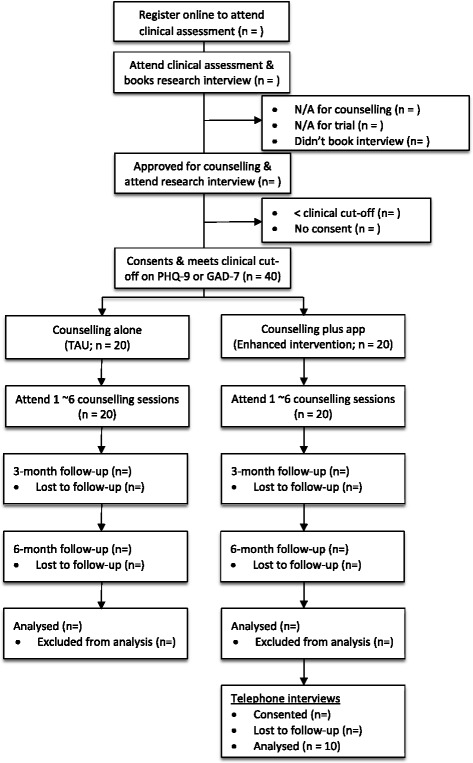
Participant flow diagram for CASELOAD feasibility trial

The feasibility trial utilises a two-arm, parallel non-randomised design comparing counselling alone (TAU) versus counselling supplemented with guided use of a well-being app and discussion of app activities (enhanced intervention) for university students experiencing anxiety or depression. This is displayed in Fig. 1. The feasibility trial was registered on the BioMed Central ISRCTN registry on 20/06/2016 under the acronym CASELOAD (Counselling plus Apps for Students Experiencing Levels Of Anxiety or Depression).

### Ethical approval

This study received ethical approval from the University of Sheffield, Department of Psychology, Research Ethics Committee (REC) on 05/01/2016 (ref: 006171).^1^ The research-informed training programme within the current study also received separate ethical approval from the University of Sheffield, Department of Psychology, REC on 17/11/2015 (ref: 006727).

### Study setting

The trial will take place at the University of Sheffield UCS which receives approximately 1,300 student referrals annually.^2^ The UCS has an ethos of supporting research and embraced the proposed feasibility trial. Extensive joint meetings took place to discuss the trial design and, in particular, to ensure that its implementation is embedded into practice with minimal disruption to the service.

### Study population

Participants are 40 help-seeking university students (aged 18 and over) who have been approved for counselling and meet moderate clinical criteria for anxiety (score ≥10 on the Patient Health Questionnaire, PHQ-9—see later section) or depression (score ≥10 on the Generalised Anxiety Disorder Scale, GAD-7—see later section). Inclusion criteria comprise (i) undergraduates (all years), (ii) postgraduates and (iii) international students. Participants will be excluded if they meet any of the following criteria: (i) present with a high risk to self or others, (ii) are currently receiving therapeutic support or (iii) have complex mental health problems beyond anxiety and/or depression.

### Recruitment

In line with routine practice, students who approach the counselling service will be assessed by a counsellor to determine their appropriateness for counselling. Students who are approved for counselling (based on clinical judgement) will be provided with a study information booklet and invited to attend a 20-min research interview onsite to determine their eligibility. Leaflets, booklets and posters will also be displayed in the waiting room to raise awareness of the trial and encourage students to volunteer. Students who attend the research interview will be asked to provide written informed consent and will be assessed for eligibility through completion of the PHQ-9 and GAD-7. Eligible participants will be allocated to either the TAU condition or enhanced intervention according to the clinical judgement of the counsellor that provided the initial assessment.

### Allocation

This study is a non-randomised feasibility trial that aims to address the acceptability of randomisation prior to planning a pilot trial in preparation for a future definitive trial. Therefore, allocation will be based on counsellors’ clinical judgement of each student’s unique situation and primary reason for approaching the service. This will inform the acceptability and feasibility of randomising for the pilot trial. Participant allocation will also depend on whether the assessing counsellor is involved in the trial (based on availability) and whether the counsellor is participating in the provision of the enhanced intervention or TAU condition. Because the enhanced intervention relies on counsellors that are committed and trained to supplement counselling with a well-being app, participants assessed by counsellors in the TAU condition will not have the opportunity to join the enhanced intervention. However, participants assessed by counsellors in the enhanced intervention may be allocated to either condition as determined by their counsellor. Similarly, participants assessed by counsellors providing the enhanced intervention for whom the app is deemed inappropriate (e.g. client presents with inappropriate/excessive technology use or risk negative exposure to online communities for self-harm) will be allocated to TAU.

Considering these design elements, allocation will depend on the following factors: (i) whether the initial clinical assessment is with a counsellor who is part of the trial; (ii) whether the assessing counsellor is allocated to provide the enhanced intervention or TAU condition; (iii) the clinical judgement of the counsellor regarding whether participating in the trial would be appropriate for the client; and (iv) the clinical judgement of the counsellor regarding which intervention would be appropriate for the client. Whilst this allocation procedure is reliant on a counsellor’s clinical judgement, it is arguably the most appropriate allocation method for a non-randomised study and it keeps the client’s welfare in the forefront. Integrating a well-being app with face-to-face counselling with students experiencing moderate anxiety or depression is a new development with limited understanding of the implications. Therefore, using clinical judgement to inform the allocation will better monitor risk and feasibility metrics which counsellors will document to inform the screening criteria of a future randomised trial.

After the routine clinical assessment, participants will attend a one-to-one research interview (approximately 20 min) to provide written informed consent and determine eligibility before potentially joining the trial. During the research interview all participants will be informed about both treatment conditions and will be asked their preference (See later section on “Treatment preference”). The preference, as well as associated reasons for preference, will be recorded in the recruitment checklist information. Irrespective of the condition, participants will not be asked to cease using any existing well-being apps, but their use will be noted in the recruitment session and explored in the exit telephone interviews. Whilst the use of existing apps may pose risk of contamination, the enhanced intervention relies on the integration of app activity within counselling and participants in the control condition will not receive guided advice on the well-being apps they may use. In addition, group differences in outcome measures can be compared before/after participants are removed for using additional well-being apps. Combined, this information will be used to inform the recruitment rate, randomisation procedure, allocation procedure and blinding for a fully powered RCT.

### Counsellors

All counsellors in the trial are accredited either by the British Association for Counselling and Psychotherapy (BACP) or the UK Council for Psychotherapy (UKCP) and are employed by the UCS. A minimum of 6 counsellors (2 control, 4 intervention) will be assigned to support the trial, both in development and delivery, and will deliver either the enhanced intervention or TAU condition based on their preference. Counsellors will also be trained by a researcher in the details specific to the intervention they are allocated to (see “Training” section below). When entering the trial, counsellors will provide a statement describing their model of practice and specific therapeutic style. The aim of collecting these statements is to improve the reporting quality when describing the therapy available, and to aid development of a clinical manual as an outcome of the feasibility trial.

Counsellors will also be provided with the BACP competency framework [14] for the University and College Counselling (UCC) context together with the most recent service clinical handbook in order to ensure best practice. Whilst these handbooks will be used to reinforce clinical competency throughout the trial, one outcome of the feasibility trial is to refine the clinical frameworks and develop a manualised training programme for delivering the enhanced intervention in a university counselling setting. For the present study, clinical practice will be reinforced throughout the trial with fortnightly team meetings with the head of service, onsite researcher and counselling team. There will also be optional daily drop-in sessions for members of the counselling team to query issues with the onsite researcher.

### Measures

**Fig. 2:**
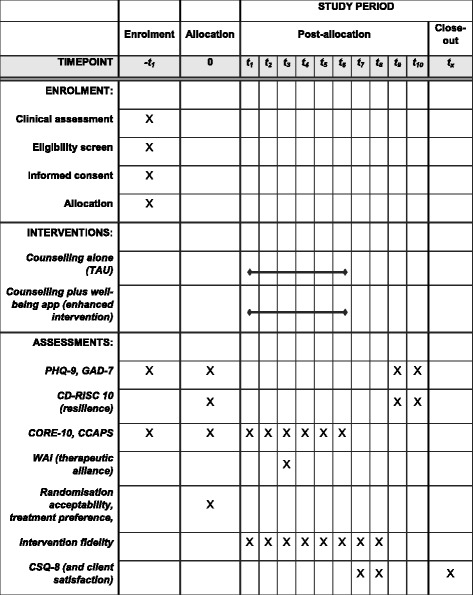
SPIRIT diagram displaying schedule of enrolment, interventions and assessments of the CASELOAD feasibility trial

The timeframe for administering measures has been summarised in Table 1 and in a Standard Protocol Items: Recommendations for Interventional Trials (SPIRIT) diagram in Fig. 2. The clinical outcome measures and primary and secondary feasibility measures have been detailed below.

**Table 1:** Summary and timeframe of measure administration

Measure	Clinical assessment	Consent session	S1	S2	S3	S4	S5	S6	Last session^a^	3-month follow-up	6-month follow-up
CORE-10	×		×	×	×	×	×	×	×		
CCAPS-62	×										
CCAPS-34			×	×	×	×	×	×	×		
PHQ-9		×								×	×
GAD-7		×								×	×
CD-RISC 10		×								×	×
WAI-12					×						
CSQ-8									×		

^a^This may be any session number beyond session 2 as the number of sessions will vary across clients and will be dependent on client-counsellor agreement

#### Clinical outcomes

##### Clinical Outcomes in Routine Evaluation (CORE-10)

The 10-item Clinical Outcomes in Routine Evaluation outcome measure [15] will be administered at the initial clinical assessment (pre-intervention) and at every counselling session, to measure changes in general psychological functioning. Items refer to the previous week and are scored on a 5-point Likert scale (0 = “not at all”; 4 = “most or all of the time”), whereby higher scores indicate higher symptom severity. CORE-10 is a shortened version of the Clinical Outcomes in Routine Evaluation - Outcome Measure [16] which has been used extensively in mental health services in the UK for over a decade. The 10-item version has been validated against CORE-OM, has been shown to be sensitive to change and can be used to determine whether a client meets membership of a clinical population (score ≥11).

##### Counseling Center Assessment of Psychological Symptoms (CCAPS)

CCAPS [17] is a measure developed in the USA specifically for the student college population and will be administered with CORE-10 at the initial clinical assessment (pre-intervention) and every counselling session to measure changes in student-specific mental health concerns. Items refer to the previous 2-week period and are scored on a 5-point Likert scale (0 = “not at all like me”; 4 = “extremely like me”), whereby higher scores indicate higher symptom severity. In addition, because CCAPS was designed for UCSs to measure student mental health, it also monitors changes in the following areas: depression, generalised anxiety, social anxiety, academic distress, eating concerns, hostility, substance abuse, family distress and suicide ideation. Within each construct, CCAPS determines clinical membership (e.g. clinical versus non-clinical) and severity (e.g. low versus elevated clinical severity), which are detailed in the CCAPS clinical guide. Finally, CCAPS has been validated in UK samples through use in UK UCS and in a recent doctoral research project [18].

##### Patient Health Questionnaire (PHQ-9) and Generalised Anxiety Disorder scale (GAD-7)

The 9-item Patient Health Questionnaire [19] and 7-item Generalised Anxiety Disorder scale [20] will be administered in the research interview, after treatment completion (3-month follow-up), and at follow-up (6-month follow-up), to measure depression and anxiety to determine eligibility. Clients who reach moderate clinical criteria for depression (score ≥10) or anxiety (score ≥10) will be invited into the trial. These measures will also be administered at 3 and 6 months after the consent date to monitor changes in symptoms. Items on PHQ-9 and GAD-7 refer to the last 2 weeks and are scored on a 4-point Likert scale (0 = “not at all”; 3 = “nearly every day”) whereby higher scores indicate higher severity. Both measures have been used widely by mental health services to measure depression and is mandatory in the Improving Access to Psychological Therapies (IAPT) initiative in the National Health Service (NHS). The purpose of using these measures in the current study is to benchmark outcomes against primary care services accessed by the general clinical population in order to allow comparisons between student and non-student clinical populations.

##### Connor-Davidson Resilience Scale (CD-RISC 10)

The 10-item Connor-Davidson Resilience Scale [21] will be administered in the research interview (pre-treatment), after completion of counselling (3-month follow-up), and at follow-up (6-month follow-up) to measure changes in resilience. Items refer to the previous month and are scored on a 5-point Likert scale (0 = “not true at all”; 4 = “true nearly all of the time”), whereby higher scores demonstrate higher resilience. By measuring resilience, the CD-RISC 10 also measures an individual’s ability to tolerate change, pressure, personal problems, negative outcomes, painful feelings and illness. The CD-RISC 10 is a short version of the original CD-RISC 25, has good internal consistency (Cronbach alpha .85), has good construct validity (e.g. resilience moderates impact of maltreatment on mental health) and has been demonstrated to have a factor structure which is more stable than CD-RISC 25 [21, 22].

###  Selection of a well-being app

There are a large variety of smartphone apps that offer tools and support for improving wellbeing via a range of common features. Whilst there are many apps to choose from they are typically based on Cognitive Behavioural Therapy (CBT) and mindfulness to offer tools for (1) tracking daily moods and behaviours, (2) reflecting on diary entries, (3) setting goals, (4) completing exercises to relax and defuse negative emotions and (5) interacting with anonymous online support communities for peer-led support. Some of the most promising well-being apps include Pacifica (http://www.thinkpacifica.com/), Headspace (https://www.headspace.com/) and Buddy App^3^. To aid the decision of selecting a well-being app for the current study, the following criteria were applied: (1) applicable to university students (e.g. providing tools to help manage social anxiety, depression, stress and general aspects of student lifestyle); (2) demonstrates potential to be integrated with face-to-face counselling; (3) available across iOS and Android platforms; (4) offers a range of features overlapping with other well-being apps; and (5) provides a promising free version to permit continued service use after the trial without financial implications.

Based on these criteria, the Pacifica app was selected and evaluated with a volunteer student sample (see next section), before it was implemented in the current feasibility trial. Whilst the app offers a free version with restricted variations of each feature (e.g. only 3 relaxation exercises compared to 8–10), the full version was used in both the evaluation study and current study to allow robust evaluation of all available features representative of other well-being apps. Furthermore, a secondary feasibility outcome will explore the added gain of using the full version compared to the free version. In both studies, a series of annual app subscriptions were purchased and provided to participants as unique gift codes. All payments were subject to the standard fee for public users and no financial incentives or waivers were provided by the Pacifica development team. Whilst the present study utilised a specific app (i.e. Pacifica), our reasoning was that this app was used in the trial as representative of well-designed apps in the field rather than being an evaluation of Pacifica per se.

### Evaluation of well-being app (Pacifica)

To aid the training, risk monitoring and intervention delivery of a well-being app in the current study, a preliminary evaluation study was conducted with the well-being app (ethical approval reference: University of Sheffield, Department of Psychology, 006727). The aims of the evaluation study were twofold: (i) to explore students’ experiences of using the well-being app to determine features that require additional support and (ii) to explore counsellors’ experiences of using the well-being app to understand how various features could compliment counselling. The student sample comprised 20 healthy volunteers (UG and PG) whereas the counsellor sample included members of the counselling team who were already scheduled to engage with the feasibility trial. Students attended a research session to learn about each app feature and were encouraged to use the app daily for 7 days. Students were instructed to use all app features once before selecting 2–3 features to use throughout the week. At the end of the week, students completed an evaluation form, inputted their app data into a spreadsheet and described their overall experience to a researcher in an interview. Based on their feedback and availability, 8 students (UG and PG with positive and negative experiences) attended a focus group to discuss their experiences and suggestions for improving the app.

The counsellor sample comprised 6 counsellors from the UCS who were scheduled to deliver the enhanced intervention condition of the feasibility trial. After attending a one-to-one research session to learn about each app feature, counsellors were instructed to use the app daily for 7 days. During this time, counsellors were advised to use all app features and to consider (1) clients who may benefit from using each feature and (2) how the app could be integrated between and within counselling sessions. At the end of the week, counsellors attended a focus group to discuss the feasibility of clients using the app alongside counselling and whether it would be feasible to review app activity during counselling sessions. Feedback from student and counsellor groups shaped the enhanced intervention and refined staff training for the current feasibility trial.

### Training

Prior to commencement of the trial, all participating counsellors will attend a one-to-one training session with a researcher to address the following: (1) knowledge of the trial research process; (2) language use for describing the trial to students; (3) counsellor expectations; and (4) areas of concern. Training sessions with counsellors in the enhanced intervention condition will additionally cover (1) willingness to audio record therapy sessions, (2) client consent to audio record therapy sessions, (3) therapeutic rationale for various app features and (4) research rationale for supplementing counselling with guided use of a well-being app. Following this, counsellors will be invited to a condition-specific training session to reduce discussion between counsellors and ensure that the enhanced intervention is delivered by counsellors who are engaged and committed to using a well-being app alongside therapy. Therefore, training with counsellors in the TAU group will only address research requirements (e.g. recruitment) and will focus on the research rationale for using an active control group and language use for recruitment.

To encourage integration of the app during counselling sessions and encourage discussion of client app activity, counsellors in the enhanced intervention will be provided with computer tablets to use during therapy sessions to review and discuss app features with clients. During training, counsellors will practice using the tablet to navigate through various app features and a range of role play exercises will be used to mimic different therapy scenarios. The tablet will also be used to audio record therapy sessions and counsellors will practice using the recording feature during training. The training session will be guided with a manual which will provide examples of how to address various scenarios as well as brief scripts to prompt counsellors during training. Script examples include the following: inviting students to book a research interview; describing the intervention in the first counselling session; confirming participant’s involvement in the study at the first counselling session; confirming permission to audio record; commencing app discussion during therapy sessions; reviewing app activity; inviting participants to use the computer tablet; and supporting participants who decide to withdraw from the trial. Counsellors will also be encouraged to make notes in their training manual to cater the examples to suit their therapeutic style.

These practical sessions aim to ensure that counsellors are confident with the technology requirements and feel at ease using the app in a therapeutic context. Therefore, counsellors will be put into pairs to practice each example and will alternate between client and counsellor roles. More specifically, when counsellors are in the role of a hypothetical client they will be asked to mimic potentially challenging behaviours they have previously experienced during sessions with their own clients. These exercises aim to challenge counsellors before they start using the app with clients. Counsellors will also have the opportunity to role-play examples with the onsite researcher throughout the trial to build confidence and refresh training. At the end of the training session, counsellors will complete a feedback form detailing their confidence in executing technical, administrative and therapeutic requirements of the trial. This information, combined with feedback from the focus group at the end of the trial, will be used to improve the manualised training for the definitive trial.

#### Technology acceptability

Despite the prevalence of technology being integrated into physical and mental health interventions, staff acceptability has been an ongoing issue and can hinder implementation [23, 24]. The current study aimed to reduce threats to staff acceptability by delivering a training programme to address various aspects of acceptability. For example, according to the technology acceptability model, there are several factors which influence technology acceptability in healthcare professionals including performance expectancy, effort expectancy, computer anxiety, computer self-efficacy and computer attitude [25]. For instance, according to Schaper and Pervan [25], performance and effort expectancy refer to the perceived ability of the technology to assist with an individual’s ability to fulfil their duty and the ease at which it can be achieved. In preparation for training in the current study, therapists used various app features daily for 1 week and were asked to consider how certain features would complement their therapeutic style. Therapists also shared their ideas in group training to inform other therapists with similar therapeutic models. Regarding effort of use, therapists were required to review a client’s app usage for a few minutes every session as a minimum but ultimately had the flexibility and responsibility to use the app as they deemed appropriate. Regarding computer anxiety and self-efficacy, in addition to the group training, therapists received ongoing one-to-one sessions with the primary researcher whom had a daily presence at the counselling service throughout the trial. Finally, regarding attitude and therapist engagement, the intervention therapists were selected based on (1) initial recommendations from the head of service and (2) expressed interest from therapists.

### Therapist effects

There has been conflicting evidence exploring the impact of therapist effects on trial outcomes and there are various methods for estimating therapist effects [26]. Nonetheless, exploring therapist effects is important in implementation studies and for considering differences in treatment delivery across therapists. Due to the underpowered sample of the current feasibility trial, therapist effects for each of the quantitative outcomes will be estimated with intra-therapist correlations.

### Counselling interventions

All participants will receive an active treatment in line with standard practice and will not be disadvantaged by participating in the trial. Participants will have access to the standard level of care at Sheffield UCS, which includes a wait period of typically 3–5 working days for the initial clinical assessment and 8–10 days between ongoing therapy sessions. This wait period varies throughout the year but the service agreement offers first contact within 10 days, and this is typically shorter than the NHS waiting times.

### Counselling (TAU)

Up to 6 sessions of face-to-face counselling will be offered to participants in line with standard practice at Sheffield UCS. Sessions will be 50 min in length and the frequency of sessions will be determined through client-counsellor discussions. If participants require more than 6 sessions, treatment will continue outside of the trial and will be supported by the counselling centre. On such occasions, trial data will only be collected up to session 6.

### Counselling supplemented with well-being app (enhanced intervention)

Up to 6 sessions of face-to-face counselling will be offered to participants in line with standard practice at the UCS. Sessions will be 50 min in length and the frequency of sessions will be determined through counsellor-client discussions. As well as the standard level of care, counselling sessions will be supplemented with discussion and guided use of a well-being app to promote engagement within and between face-to-face sessions. Clients and counsellors will have the opportunity to use the app on a computer tablet during counselling sessions to facilitate discussion and to aid the decision process for setting goals and reviewing client progress. Through these discussions, counsellors will review client app activity and guide them through various app features to decide which activities would be beneficial to use between face-to-face sessions. App features may include (1) daily behaviour tracking for mood, sleep, exercise, relationships, hygiene, water/caffeine/alcohol consumption, medication use and time spent outside; (2) reflective thinking through guided CBT, thought journaling, mindfulness and positive visualisation; (3) guided relaxation with breathing, meditation and body scan exercises; (4) peer-led support through anonymous online communities and private groups; and (5) setting/tracking short-term and long-term goals.

The app will provide daily prompts to encourage participants to log their mood/behaviour, but completion of various exercises relies on participants deciding when to use a feature, for example at the suggestion of their counsellor. Counsellors will also encourage participants to prepare for each face-to-face counselling session by reflecting on their diary entries and deciding on what they would like to address in the session. This reflective exercise may also occur at the start of each therapy session for participants who prefer to reflect on their activities with the support of their counsellor. During face-to-face sessions, counsellors will be encouraged to review app activity by asking participants to access their app account on the computer tablet, discuss participant’s reflections and progressively adjust goals or exercises where appropriate.

#### Audio recording of sessions

Counselling sessions in the enhanced intervention will be audio-recorded, with participant consent, using the tablet in order to be more discrete than traditional recording equipment. Written consent for recording sessions will initially be sought during the research interview when participants join the trial. However, verbal consent will also be sought by counsellors at the start of each session to allow participants to opt-out of recording a particularly distressing counselling session. Sessions in the TAU condition will not be audio recorded to align with standard practice and because analysis is specific to discussing app activity, which is dependent on the enhanced intervention.

### Primary feasibility measures

The yield of the feasibility trial is a series of specific primary and secondary outputs relating to a range of components that will inform the definitive trial. The primary outputs are recruitment, treatment preference, randomisation acceptability, treatment satisfaction and completion rate of follow-up measures. The secondary outputs are: app usage, intervention fidelity, client characteristics, therapeutic alliance and academic coping. Each of these primary and secondary outputs is described next.

#### Recruitment

The recruitment period for a definitive trial will be estimated from the current study by exploring the required time needed to reach 40 participants, whilst also considering the participant drop-out rate and counsellor availability. Seasonal service demands will also be explored to distinguish peak service demand and to advise on the optimal time of year to implement a definitive trial. Service demand will be assessed by comparison of the annual reports provided from the service.

#### Treatment preference

During the research interview and prior to treatment allocation, participants will be informed about the two available treatment conditions and will be asked to indicate their preferred condition. Participants will also provide a primary reason for their decision before being informed of their treatment allocation. Participants who choose a condition incongruent with their allocation will be asked if the outcome affects their decision to join the trial. This information will be used to inform potential bias from participants being allocated to their preferred condition.

#### Randomisation acceptability

Once participants state their treatment preference and are informed of their actual treatment allocation, they will be asked whether being randomised to that condition increases the probability of them withdrawing from the study. This information, combined with the recruitment metrics, will be used to estimate the recruitment period and inform whether randomisation in a definitive trial would negatively impact on uptake.

#### Treatment satisfaction

Participant treatment satisfaction will be assessed with the 8-item Client Satisfaction Questionnaire (CSQ-8) [27], which will be emailed to participants the day after their last counselling session. CSQ-8 items refer to a client’s overall service experience and are rated on a 4-point Likert scale (1 = “quite dissatisfied”; 5 = “very satisfied”) whereby higher scores indicate greater satisfaction. Many counselling services report on client satisfaction to allow comparison to other services and to ensure that services respond to client feedback. Capturing client satisfaction will also allow comparisons between treatment conditions to explore whether the enhanced intervention had a positive or negative impact on a participant’s service experience. A sub-sample of participants’ service experiences will also be explored through telephone interviews after counselling completion. Finally, counsellor satisfaction in the delivery of the enhanced intervention will be assessed through a focus group once all participants have completed counselling.

#### Completion rate of follow-up measures

Completion rates will be assessed through the number of participants providing complete data for the 3-month follow-up measures (from consent date), 6-month follow-up measures and telephone interviews. The telephone interviews will also be used to ask participants how to optimise the follow-up response rate and maintain contact. Together, this information will be used to estimate the expected response rate and inform the design of a definitive RCT.

### Secondary feasibility measures

#### App usage

App usage during counselling sessions will be assessed through audio recordings to determine how various features are discussed between participants and counsellors. The discussion of clients’ app usage to monitor behaviours, thoughts, emotions and therapeutic exercises is an essential component of using feedback in therapy and in integrating the well-being app with counselling. Therefore, analysis of app discussion aims to identify dominant app features, inform potential moderators of therapeutic outcomes in the definitive trial and evaluate intervention fidelity (discussed below). Analysis of app discussion will also consider the added gain of using the purchased app version over the free version, by categorising discussion by features associated with either version of the app. A final exploration of app discussion will match app features with context-specific benefits, client characteristics and potential risk.

Participant app usage between counselling sessions will be addressed in follow-up telephone interviews to inform the acceptability of using a well-being app alongside counselling, and exploring the timeframe for how long the well-being app was useful for participants. Participant usage of the app overtime will also be explored through app data (e.g. log in times, duration of time spent using each feature), but this will be dependent on the availability of data and on participant consent to access app data.

#### Intervention fidelity

Intervention fidelity will be assessed through counselling audio recordings, a focus group with counsellors and telephone interviews with participants. Counselling recordings will be anonymised during transcription and assessed by two reviewers, the onsite researcher (unblinded) and an independent blinded researcher, to permit analysis of inter-rater reliability. A checklist will be provided to score the transcript content which will include the following themes, separately for counsellors and participants: (1) number of times app discussed; (2) duration of app discussion; (3) whether the tablet was used to view app activity; (4) whether there was reason to adjust app usage; (5) whether different app features were advised; and (6) whether there was a missed opportunity to discuss an app feature. Audio recordings will also be assessed against the BACP UCC competency framework to determine clinical competency and to develop the framework for the definitive trial. Finally, intervention fidelity will be assessed in the counsellor focus group and participant interviews to explore challenges of integrating the app with counselling and to provide potential solutions.

#### Client characteristics

Client characteristics will be assessed collectively from (i) intake demographic data, (ii) counsellor notes from the initial clinical assessment, (iii) counsellor session notes and (iv) participant interviews. Combined, this information will be used to develop a client checklist and brief clinical guide to aid decision making on client appropriateness for using a well-being app. This guide is anticipated to inform the inclusion criteria for the definitive trial and will be shared with other UK UCSs interested in offering well-being apps to their students.

#### Therapeutic alliance

Therapeutic alliance will be assessed through the Working Alliance Inventory-Short Form [28] at the end of session 3 of counselling. WAI is a 12-item self-report measure completed separately by the counsellor and their client. Items refer to current views on the counsellor/client and are rated on a 5-point Likert scale (1 = “strongly disagree”; 5 = “strongly agree”) whereby higher scores indicate stronger therapeutic alliance. Items also provide scores on three distinct components of therapeutic alliance including (1) agreement of therapy tasks, (2) agreement of therapeutic goals and (3) presence of an affective bond between clients and counsellors. These therapeutic factors will be compared across the enhanced intervention and TAU conditions to explore differences in therapeutic alliance and inform potential mediators of clinical outcomes to be tested in a definitive trial.

#### Academic coping

To complement the academic distress measure on the CCAPS [17], participants will be asked about their ability to cope academically during follow-up telephone interviews. During the interviews, participants will be asked whether their mental health has affected their studies (or vice versa) and whether they believe that counselling has contributed to their ability to cope academically. These findings will be used to explore the potential contribution UCSs have on academic coping, and will inform outcome assessment for a definitive trial.

### Managing risk and adverse events

All stages of the feasibility trial will take place at the UCS and participants will have immediate access to professional mental health support. Counsellors involved in the trial will also have access to professional mental health support through the duty counsellor in line with standard practice. Efforts have been made to reduce risk in the current study by ensuring that the design, training and delivery of interventions are informed by clinical judgement, and clinical competency is reinforced by the BACP competency framework and UCS clinical handbook. Furthermore, all decisions concerning participant allocation are informed by their assessing counsellor (see “Allocation” section). Counsellor training will also address participant withdrawal and how to report risk. In either event, every individual involved with the trial (e.g. admin, clinical and research) will be informed to report to the duty counsellor allocated at the start of each day. The reporting of adverse events will be recorded through (1) onsite researcher notes throughout trial, (2) therapist clinical notes from triage, (3) therapist notes from counselling and (4) duty therapist clinical notes. In line with the service clinical handbook, adverse events will be reported to the duty therapist and recorded electronically on the service’s secure clinical scheduling system.

### Data management

A primary researcher (author EB) will be selected to oversee all stages of the feasibility trial and will include the following responsibilities: (1) maintain primary contact with staff at the UCS; (2) deliver counsellor training; (3) deliver research interview with participants; (4) administer and score PHQ-9 and GAD-7 to determine participant eligibility; (5) offer ongoing support to counsellors by maintaining a physical presence at the UCS; (6) offer technical support of computer tablets; and (7) handle trial data from paper and electronic sources. As the primary researcher will oversee all stages of the feasibility trial and will handle all trial data, it was decided not to form a Data Monitoring and Ethics Committee; however, the primary researcher will regularly update with the head of service at the participating trial centre and authors AM and MB as research supervisors for the feasibility trial. Methods of planned data management have been approved by the trial sponsor REC, and have been implemented to be predominantly electronic to avoid human error and optimise data security.

Part of data management will require storing participant consent forms and documents from the research interview in a securely locked filing cabinet at the UCS. The filing cabinet will only be accessed by the primary researcher and the clinical team, if necessary. Therapy audio recordings from computer tablets will be immediately uploaded to an encrypted file (via UCS encrypted Wi-Fi) on the UCS computer system, and will not be stored on computer tablets. This process is automatic and will be triggered when the audio recording app is stopped. Only the primary researcher will have access to the encrypted folder and recordings will be anonymised upon transcription. The remaining sources of clinical data (e.g. from questionnaires) will be administered online via unique survey links emailed to participants. Survey data will only be accessed through a secure account log-in which only the primary researcher will have access to. All data will be backed up on an encrypted external hard drive, accessed only by the primary researcher. Data will be stored in a Microsoft Access database on two encrypted USBs handled by the primary researcher.

### Statistical analyses

#### Quantitative analysis

Analyses will be predominantly descriptive to characterise the study population and outline various feasibility metrics including recruitment rate, treatment preference, randomisation acceptability, treatment satisfaction and completion at follow-up. Whilst the sample size is not powered to detect significant differences between the TAU and enhanced intervention groups, data will be used to summarise outcomes from both groups to reveal preliminary trends and inform the design of the pilot trial from which estimates of effect and sample sizes will be calculated. Group comparisons will also be made between the demographic and baseline clinical measures to characterise the groups when they enter the trial. Determining potential baseline differences between the groups will inform whether the allocation procedure, which was dependent on clinical judgement, unintentionally created differences between the groups. Establishing these differences, or lack thereof, will further inform the potential group differences in clinical outcomes at the end of the feasibility trial. Outcome data will include the baseline prevalence and subsequent changes in depression (PHQ-9), anxiety (GAD-7), psychological functioning (CORE-10), student-specific mental health concerns (CCAPS) and emotional resilience (CD-RISC 10). Group summaries will also compare levels of therapeutic alliance (WAI) and treatment satisfaction (CSQ-8) in order to inform preliminary differences across treatments. No interim analysis will be performed; analysis will commence after completion of the 6-month follow-up. The distribution, variance and skewness of data will be initially explored to determine whether data should be described with parametric or non-parametric methods. Parametric descriptive statistics will include total score, mean, standard deviation, min, max and range. Non-parametric descriptive statistics will include total score, median, confidence intervals and inter-quartile ranges. Quantitative analyses will be performed with SPSS statistical software (version 22.0).

Approximately 40 h of therapy is anticipated to be recorded, transcribed and analysed. The conservative estimation for recordings considers several factors including the following: (1) although participants will be offered ~6 counselling sessions, the services’ median number of attended sessions is 2 and 4; (2) participants (and therapists) may decide not to record a session where the participant is particularly distressed; (3) trial budget available to fund transcription; and (4) feasibility trials are not required to be powered to detect significant effects but to provide sufficient preliminary indicators. Whilst data from the therapy recordings is qualitative in nature, they will be analysed with “quantitative” content analysis [29] whereby sessions will be scored to indicate intervention fidelity and implementation success. These scores will be achieved by using a checklist developed from the training materials to rate the extent to which therapists delivered the new intervention (see “Intervention fidelity” section). A random sample of 15 therapy hours will be assessed by an independent researcher, blind to the aims of the study, to permit analysis of inter-rater reliability.

#### Qualitative analysis

A number of feasibility factors will be explored qualitatively through counsellor clinical notes, a therapist focus group, and participant exit interviews; to explore the feasibility, acceptability and potential implications of supplementing counselling with a well-being app. The clinical notes will be extracted from the sessional notes taken as part of routine practice, except counsellors will additionally reflect on their experience of integrating the app and how the app fitted with their client/therapy style. These experiences will be explored in more detail at the end of the trial through a one-off therapist focus group. The participant exit interviews will take place once clients finish counselling and thus may occur throughout the trial depending on how early clients were recruited and how many counselling sessions they agreed to have with their therapist. The aims of the exit interviews are threefold: (1) to capture clients’ experiences of the new intervention (and indirectly inform intervention fidelity); (2) to distinguish areas of improvement for research design; and (3) to identify whether counselling contributed to their ability to cope at university. Data from the client interviews, therapist focus group and clinical notes will be analysed flexibly and explorative with thematic analysis to allow themes to emerge from the data and to allow comparisons across different data sources [30]. By exploring themes across various data sources, the current study aimed to provide a rounded and comprehensive account for the following: (1) how well the app was integrated into counselling (according to clients, therapists and researchers) and (2) the potential risks/benefits of integrating an app with counselling, if implementation is successful. Qualitative data will be analysed with NVivo (version 11).

## Discussion

Using a mixed-methods approach incorporating quantitative and qualitative data, this study will address a range of factors concerning the feasibility of supplementing counselling with guided use of a well-being app for university students experiencing anxiety or depression. Through this exploration, the primary feasibility outcome will determine whether it is possible to incorporate, review, and discuss participant app usage during face-to-face counselling sessions in a manner that is potentially beneficial to therapeutic outcomes. For this reason, the study design offers an active control to mimic standard practice and permit preliminary comparisons to be made with the enhanced intervention. By comparing the enhanced intervention with a condition mimicking standard treatment (TAU), this study will shed light on whether potential differences in group outcomes could be accountable by the addition of a well-being app alongside standard care. However, whilst this feasibility trial is not powered to detect significant differences between group outcomes, it will allow the identification of trends to inform the hypotheses for a definitive RCT. Furthermore, combined analysis on counselling recordings, interviews and a focus group on the enhance intervention will reveal possible treatment mechanisms which can then be assessed quantitatively in a fully powered trial.

A key goal of this feasibility trial is to explore the feasibility of the planned processes and document the issues that arise throughout the training, implementation, delivery and evaluation of research design and overall intervention. Therefore this study focuses on demonstrating the feasibility of offering a new treatment option to university students, and to review the potential implications for improving therapeutic outcomes. To address the first requirement, primary feasibility metrics will record: recruitment rate, treatment preference, randomisation acceptability, treatment satisfaction and completion of follow-up measures. However, if the new intervention is shown to be feasible, it is also important to understand the potential risks, implications and mechanisms to be explored in a definitive trial. Therefore, the secondary feasibility metrics will address app usage, intervention fidelity, therapeutic alliance and a range of clinical outcome measures monitoring mental health symptoms specific to university students. With anxiety and depression as the two most prevalent mental health concerns in students, participant eligibility will be determined through two clinical diagnostic tools, PHQ-9 and GAD-7, used widely in psychological services.

The planned enhanced intervention combines the benefits of face-to-face counselling with the flexibility of guided self-help, for university students experiencing anxiety and depression. By combining two treatment options which are typically offered separately, the current study aims to address a number of challenges USCs experience. The most prominent challenges have been supporting a growing student population with short-term therapy that fits within the academic calendar. Through these challenges, UCSs have experienced increased waiting lists, higher rates of treatment drop-out and more demand for support during evenings, weekends and university holidays. Therefore, by combining face-to-face counselling with guided self-help support and behavioural tracking tools on a mobile app, the current study aims to demonstrate a preliminary impact on engagement, drop-out and therapeutic outcomes. Furthermore, by encouraging self-help tools between face-to-face sessions, the current study aims to offer ongoing support to students and optimise therapeutic time between clients and counsellors. If this new treatment option is shown to be feasible, the current study has potential to encourage flexible working styles and enhance existing face-to-face time without necessarily requiring more therapy sessions. These opportunities, amongst improving training, will be the primary aims of a definitive RCT following a pilot trial to be planned beyond the current feasibility study.

## Additional file

Additional file 1: Trial ethics approval letter. (PDF 29 kb)

## Abbreviations

BACP: British Association for Counselling and Psychotherapy
CASELOAD: Counselling plus Apps for Students Experiencing Levels of Anxiety or Depression
CBT: Cognitive behavioural therapy
CCAPS: Counseling Center Assessment for Psychological Symptoms
CD-RISC 10: Connor Davidson Resilience Scale (10-item version)
CD-RISC 25: Connor Davidson Resilience Scale (25/full-item version)
CORE-10: Clinical Outcomes in Routine Evaluation-10
CORE-OM: Clinical Outcomes in Routine Evaluation Outcome-Measure
CSQ-8: Client Satisfaction Questionnaire
GAD-7: Generalised Anxiety Disorder Scale
HE: Higher education
NHS: National Health Service
PG: Postgraduate
PHQ-9: Patient Health Questionnaire
RCT: Randomised controlled trial
REC: Research ethics committee
TAU: Treatment as usual
UCC: University and college counselling
UCS: University counselling service
UG: Undergraduate
UK: United Kingdom
UKCP: United Kingdom Council for Psychotherapy
WAI: Working Alliance Inventory

## Footnotes

1 The confirmation letter from REC has been submitted as Additional file 1.

2 Based on referrals across 2011/12-2013/14 academic periods.

3 Buddy App was availble during the original app search, but is no longer publically available.

## References

[1] Osborne M (2003). Policy and practice in widening participation: a six country comparative study of access as flexibility. Int J Lifelong Ed.

[2] Holm-Hadulla RM, Koutsoukou-Argyraki A (2015). Mental health of students in a globalized world: prevalence of complaints and disorders, methods and effectivity of counseling, structure of mental health services for students. Ment Health Prev.

[3] Mohr DC, Burns MN, Schueller SM, Clarke G, Klinkman M (2013). Behavioral intervention technologies: evidence review and recommendations for future research in mental health. Gen Hosp Psychiatry.

[4] Clarke G, Yarborough BJ (2013). Evaluating the promise of health IT to enhance/expand the reach of mental health services. Gen Hosp Psychiatry.

[5] Kitzrow MA (2003). The mental health needs of today’s college students: challenges and recommendations. NASPA J.

[6] Eisenberg D, Golberstein E, Gollust SE (2007). Help-seeking and access to mental health care in a university student population. Med Care.

[7] Randall EM, Bewick BM (2015). Exploration of counsellors’ perceptions of the redesigned service pathways: a qualitative study of a UK university student counselling service. Br J Guid Couns.

[8] Krägeloh CU, Czuba KJ, Billington DR, Kersten P, Siegert RJ (2015). Using feedback from patient-reported outcome measures in mental health services: a scoping study and typology. Psychiatric Serv.

[9] Kendrick T, El-Gohary M, Stuart B, Gilbody S, Churchill R, Aiken L, Bhattacharya A, Gimson A, Brütt AL, Jong K, Moore M (2016). Routine use of patient reported outcome measures (PROMS) for improving treatment of common mental health disorders in adults. Cochrane Database of Syst Rev.

[10] Swan M (2012). Health 2050: the realization of personalized medicine through crowdsourcing, the quantified self, and the participatory biocitizen. J Perinat Med.

[11] McCrone P (2004). Cost-effectiveness of the computerised cognitive-behavioural therapy for anxiety and depression in primary care: randomised controlled trial. Br J Psychiatry.

[12] Barak A, Hen L, Boniel-Nissim M, Shapira N (2008). A comprehensive review and a meta-analysis of the effectiveness of internet-based psychotherapeutic interventions. J Technol Hum Serv.

[13] Cavanagh K, Millings A (2013). (Inter) personal computing: the role of the therapeutic relationship in e-mental health. J Contemp Psychother.

[14] Hill A, Roth T. Competences required to deliver effective counselling in further education. BACP. 2016. http://www.bacp.co.uk/ethics/competences_and_curricula/. Accessed 3 Nov 2016.

[15] Barkham M, Bewick B, Mullin T, Gilbody S, Connell J, Cahill J, Mellor-Clark J, Richards D, Unsworth G, Evans C (2013). The CORE-10: a short measure of psychological distress for routine use in the psychological therapies. Couns Psychother Res.

[16] Barkham M, Margison F, Leach C, Lucock M, Mellor-Clark J, Evans C, McGrath G (2001). Service profiling and outcomes benchmarking using the CORE-OM: toward practice-based evidence in the psychological therapies. Clinical Outcomes in Routine Evaluation-Outcome Measures. J Consult Clin Psychol.

[17] Locke BD, Buzolitz JS, Lei PW, Boswell JF, McAleavey AA, Sevig TD, Hayes JA (2011). Development of the Counseling Center Assessment of Psychological Symptoms-62 (CCAPS-62). J Couns Psychol.

[18] Broglia E, Millings A, Barkham M. The Counseling Center Assessment of Psychological Symptoms (CCAPS‐62): Acceptance, feasibility, and initial psychometric properties in a UK student population, Clin Psychol Psychother. 2016. doi: 10.1002/cpp.2070 10.1002/cpp.2070 28124512

[19] Spitzer RL, Kroenke K, Williams JBW (1999). Validation and utility of a self-report version of PRIME-MD: the PHQ primary care study. JAMA.

[20] Spitzer RL, Kroenke K, Williams JBW, Lowe B (2006). A brief measure for assessing generalized anxiety disorder: the GAD-7. Arch Intern Med.

[21] Campbell-Sills L, Stein MB (2007). Psychometric analysis and refinement of the Connor-Davidson Resilience Scale (CD-RISC): validation of a 10-item measure of resilience. J Trauma Stress.

[22] Connor KM (2003). Development of a new resilience scale: the Connor-Davidson Resilience Scale (CD-RISC). Depress Anxiety.

[23] Waller R, Gilbody S (2009). Barriers to the uptake of computerized cognitive behavioural therapy: a systematic review of the quantitative and qualitative evidence. Psychol Med.

[24] Luxton DD, McCann RA, Bush NE, Mishkind MC, Reger GM (2011). mHealth for mental health: integrating smartphone technology in behavioral healthcare. Prof Psychol Res Prac.

[25] Schaper L, Pervan G (2007). A model of information and communication technology acceptance and utilisation by occupational therapists. Stud Health Technol Inform.

[26] Lutz W, Leon SC, Martinovich Z, Lyons JS, Stiles WB (2007). Therapist effects in outpatient psychotherapy: a three-level growth curve approach. J Couns Psychol.

[27] Attkisson CC, Zwick R (1982). The Client Satisfaction Questionnaire: psychometric properties and correlations with service utilization and psychotherapy outcome. Eval Prog Plan.

[28] Hatcher RL, Gillaspy JA (2006). Development and validation of a revised short version of the Working Alliance Inventory. Psychother Res.

[29] Satu E, Kyngäs H (2008). The qualitative content analysis process. J Adv Nurs.

[30] Braun V, Clarke V (2006). Using thematic analysis in psychology. Qual Res Psychol.

