# Supplementary material for: Comparing counselling alone versus counselling supplemented with guided use of a well-being app for university students experiencing anxiety or depression (CASELOAD): protocol for a feasibility trial
Source: Pilot Feasibility Stud. 2017 Jan 23;3:3. doi: 10.1186/s40814-016-0119-2 (PMC5256558; doi:10.1186/s40814-016-0119-2)
Supplement: Additional file 1: — Trial ethics approval letter. (PDF 29 kb) [file 40814_2016_119_MOESM1_ESM.pdf]

Downloaded: 05/01/2016
Approved: 05/01/2016

Emma Broglia
Registration number: 140234406
Psychology
Programme: PhD Psychology (ft)

Dear Emma

PROJECT TITLE: A feasibility trial comparing embedded university counselling versus counselling supplemented with a well-being app for students with anxiety or depression
APPLICATION: Reference Number 006171

On behalf of the University ethics reviewers who reviewed your project, I am pleased to inform you that on 05/01/2016 the above-named project was **approved** on ethics grounds, on the basis that you will adhere to the following documentation that you submitted for ethics review:

- University research ethics application form 006171 (dated 14/12/2015).
- Participant information sheet 1014309 version 1 (14/12/2015).
- Participant information sheet 1014312 version 1 (14/12/2015).
- Participant information sheet 1014311 version 1 (14/12/2015).
- Participant information sheet 1014310 version 1 (14/12/2015).
- Participant consent form 1014304 version 1 (14/12/2015).
- Participant consent form 1014325 version 1 (14/12/2015).

The following optional amendments were suggested:

One reviewer commented on a methodological issue that you might want to consider: "I do wonder if the non-random allocation of participants will compromise the ability of the study to inform on the effectiveness of the intervention and also will limit its usefulness in informing the design of an RCT that presumably will involve randomly allocating participants" However, I think that this study should be approved as the ethical issues are dealt with soundly

If during the course of the project you need to deviate significantly from the above-approved documentation please inform me since written approval will be required.

Yours sincerely

Thomas Webb
Ethics Administrator
Psychology
